# Excisional Treatment of Cervical Dysplasia in Australia 2004–2013: A Population-Based Study

**DOI:** 10.1155/2016/3056407

**Published:** 2016-04-30

**Authors:** Gregory Robertson, Stephen J. Robson

**Affiliations:** ^1^University of New South Wales, School of Women's and Children's Health, Randwick, Sydney, NSW 2031, Australia; ^2^Centre for Gynaecological Oncology, Royal Hospital for Women, Randwick, Sydney, NSW 2031, Australia; ^3^Australian National University Medical School, Garran, ACT 2605, Australia

## Abstract

*Background*. Excisional treatment of preinvasive cervical dysplasia has been associated with adverse pregnancy outcomes. We aimed to examine trends in the rate of excisional treatment in reproductive age women in the era of HPV vaccination.* Methods*. National data for Australia regarding histological diagnoses of cervical dysplasia and excisional treatment for the period from 2004 to 2013 inclusive were obtained from two datasets and used to calculate age-stratified incidence rates of excisional treatment and of excisional treatments per diagnosis of dysplasia.* Results.* The incidence of low-grade squamous dysplasia fell in all age groups, while the incidence of high-grade dysplasia fell in the 20-to-24-year group but rose slightly for older age groups. The rate of excisional treatment fell in women aged under 35 but there was no significant change for women 35 years or older. The rate of all excisional treatments (loop excision + cone biopsy) per high-grade diagnosis (CIN2 + CIN3 + adenocarcinoma* in situ*) fell across all three age-bands in both datasets.* Conclusion*. To ensure that the use of excisional treatment is appropriate, with lower rates for younger HPV-vaccinated women, close surveillance, audit, and ongoing education will be required.

## 1. Introduction

Cervical cancer is the third most common gynaecological cancer in Australia, and virtually all cases result from infection with human papilloma virus (HPV) [1, 2]. Over the life of the Australian National Cervical Screening Program (NCSP) there has been a 50% reduction in both the incidence and mortality of cervical cancer, and most new cases occur in women who have not fully participated in the program [1, 3]. To further reduce the incidence of cervical cancer, a government-funded population-based National HPV Vaccination Program (NHVP) commenced in Australia in 2007. This program aimed to deliver a course of three vaccinations of the quadrivalent HPV vaccine* Gardasil™* to girls aged 12 to 13 years, with a two-year “catch-up program” for 13- to 18-year-old girls through schools and a two-year “catch-up program” for women aged 18 to 26 years through general practices and community-based programs. The vaccination coverage for school-aged girls is now 70% [3].

In early 2009, two years after the NHVP had commenced, the Royal Australian and New Zealand College of Obstetricians and Gynaecologists set up a quality improvement program in colposcopy (*c-Quip*) with the aim of improving care for women with screen-detected abnormalities [4]. One of the key drivers of the* c-Quip* program is that the number of women who are treated with a low-grade eventual diagnosis on pathology is minimised. This audit standard was chosen due to concern regarding the use of excisional treatment in young women with a good prognosis. We set out to examine the trends in excisional treatment in Australia over the last decade and whether the NHVP or* c-Quip* program had been associated with alterations in the incidence of cervical excisional treatment in young women.

## 2. Materials and Methods

Data were obtained from four mandated national data collections to develop a single dataset for this study. To provide a population denominator, census-derived point estimates of the age-specific female population in Australia for the period 2004 to 2013 were obtained from the Australian Bureau of Statistics (ABS). Data regarding the number of histological diagnoses of cervical dysplasia were obtained from the Australian Institute of Health and Welfare (AIHW). The AIHW dataset reports statistics from the cervical screening registers of each state and territory, compiled into the national dataset by the AIHW for purposes of national monitoring and audit. Complete data (including endocervical diagnoses) were available from 2004 to 2013 inclusive [5].

Australia has no overarching dataset regarding all procedures in both inpatient and outpatient (office) settings. To capture the most complete dataset possible, data regarding procedures were obtained from two sources. The first source was the AIHW national procedural dataset, collected under the auspice of the Australian Health Ministers' Advisory Council (AHMAC) through the National Health Information Agreement. These data are collected as specified in the National Minimum Data Sets relating to hospitals and day procedure facilities, pooling data supplied by state and territory health authorities. It is important to recognize that these data provide information only about procedures performed on admitted patients in hospitals and day surgery facilities. Validation studies of the AIHW dataset have reported 99.5% agreement with “true” morbidity (kappa 0.86) [6].

To cover excisional procedures performed in nonadmitted patients, our other source of procedural information was Medicare data from the Australian National Medical Benefits Scheme (MBS) billing dataset. The MBS billing data report procedural services that are performed by a registered provider for services that qualify for Medicare benefit and for which a claim has been processed by Medicare Australia. Thus, these are complimentary data to the AIHW, capturing both inpatient procedures and outpatient (rooms) procedures billed through Medicare. The MBS dataset does not include services provided by hospital doctors to public patients in public hospitals (procedures captured in the AIHW dataset) or services that qualify for a benefit under the Department of Veterans' Affairs (DVA) National Treatment Account. The AIHW and MBS datasets are complementary and obviously not mutually exclusive, so we separately analysed each dataset.

The extracted data were entered into Excel*™* spreadsheets. Analysis of variance was undertaken using regression with calculation of *R* and adjusted *R* ^2^ (*aR* ^2^) values, and comparisons were made using 2 × 2 tables to calculate odds ratios and 95% confidence intervals, with chi-square to calculate *p*-values. This study received approval from the Australian National University Human Research Ethics Committee (protocol 2015/347).

## 3. Results

**Figure 1:**
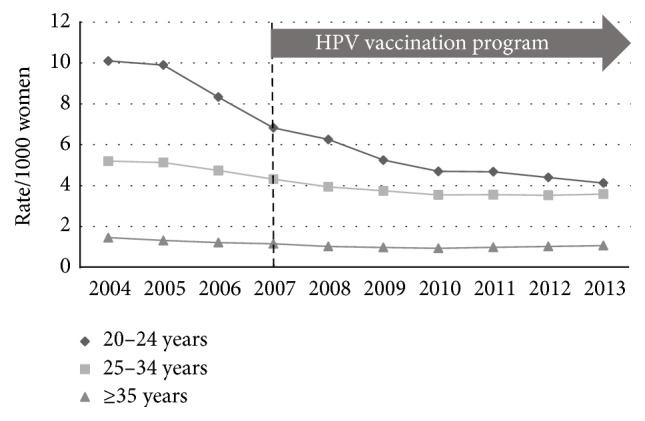
Histological diagnoses of low-grade epithelial abnormality (LGEA) per 1000 of female population, age-stratified, for the period 2004–2013.

**Figure 2:**
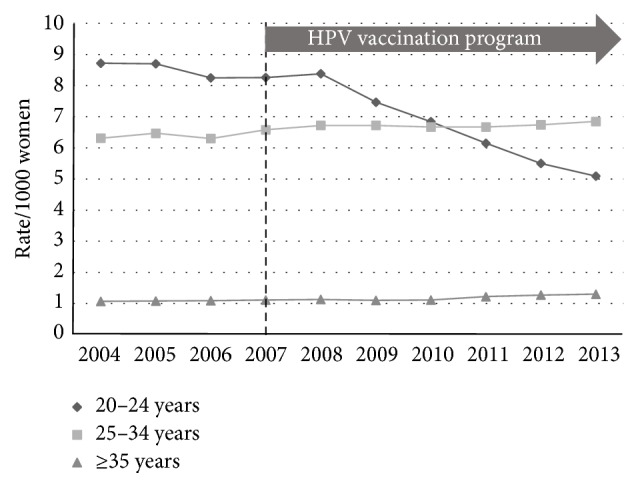
Histological diagnoses of high-grade epithelial abnormality (HGEA) per 1000 of female population, age-stratified, for the period 2004–2013.

**Figure 3:**
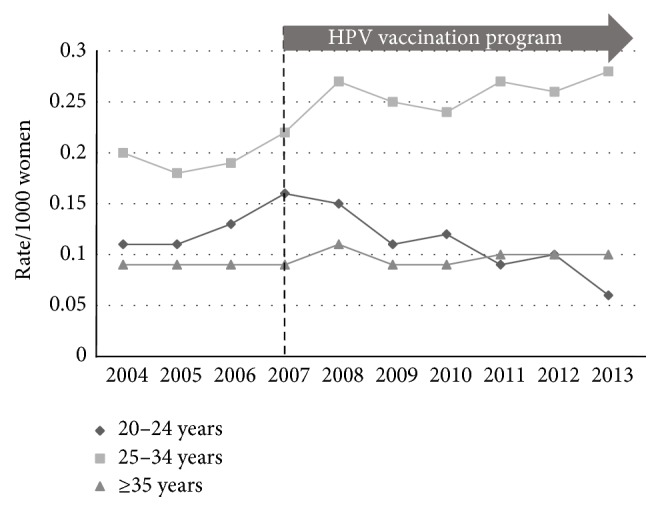
Histological diagnoses of adenocarcinoma* in situ* per 1000 of female population, age-stratified, for the period 2004–2013.

National data regarding the incidence of low-grade (HPV effect + CIN1), high-grade (CIN2 + CIN3), and endocervical dysplasia (adenocarcinoma* in situ*) for the period from 2004 to 2013 inclusive were used with the ABS population estimates to calculate age-stratified incidence rates (number of histologically confirmed cases per 1000 women) between the prevaccination epoch (2004–2007) and the postvaccination epoch (2008–2013). The incidence of low-grade change fell across all three age-bands (Figure 1): 20 to 24 years (*p* < 0.005); 25 to 34 years (*p* < 0.005); and 35 years or older (*p* < 0.005). The incidence of high-grade change fell in the 20-to-24-year group (*p* < 0.005) but rose slightly for the older age groups (Figure 2): 25 to 34 years (*p* < 0.005) and 35 years and older (*p* < 0.005). The incidence of glandular dysplasia was unchanged for the age group 20 to 24 years (*p* = 0.09), increased for the age group 25 to 34 years (*p* < 0.005), and remained unchanged for the age group 35 years or older (*p* = 0.15) (Figure 3).

**Figure 4:**
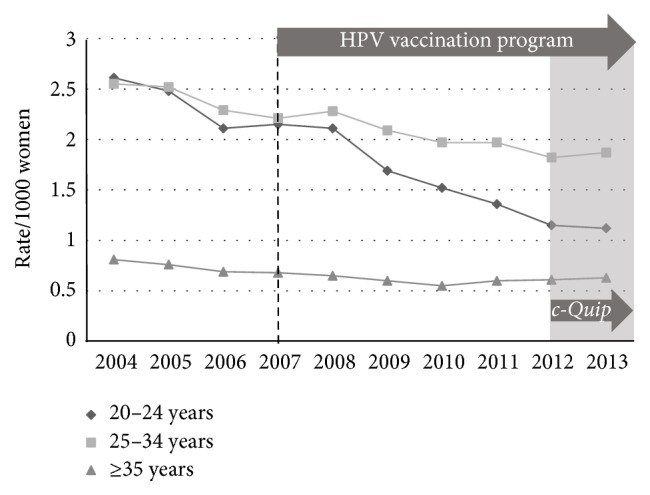
Medicare MBS billing data showing all excisional treatments (loop excision + cone biopsy) per 1000 of female population, age-stratified, for the period 2004–2013.

**Figure 5:**
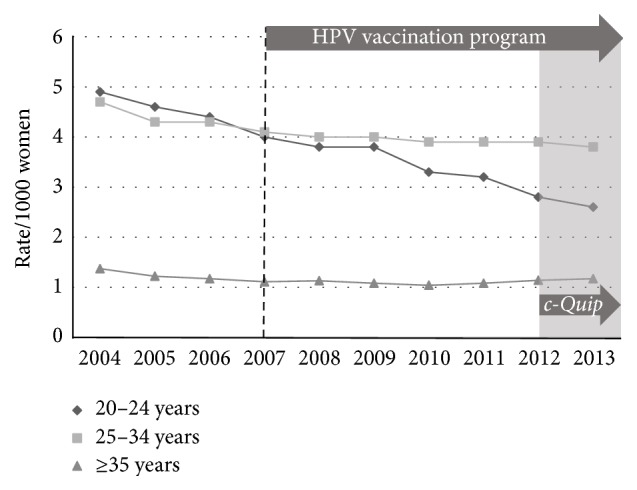
AIHW procedures data showing all excisional treatments (loop excision + cone biopsy) per 1000 of female population, age-stratified, for the period 2004–2013.

The MBS billing dataset was the smaller of the two, recording 82521 excisional treatments during the ten-year study period. The AIHW procedural dataset contained 153421 excisional procedures over the same period, 86% more than the MBS. Age-stratified incidence rates for all excisional treatments (loop excision + cone biopsy) were calculated separately for the MBS and AIHW datasets. In the MBS dataset the incidence rate of excisional treatment fell significantly in all age groups across the study period (Figure 4). The AIHW dataset showed significant reductions for the age groups 20 to 24 years (*p* < 0.005) and 25 to 34 years (*p* < 0.005); however, there was no significant change for women 35 years or older (*p* = 0.06) (Figure 5).

**Figure 6:**
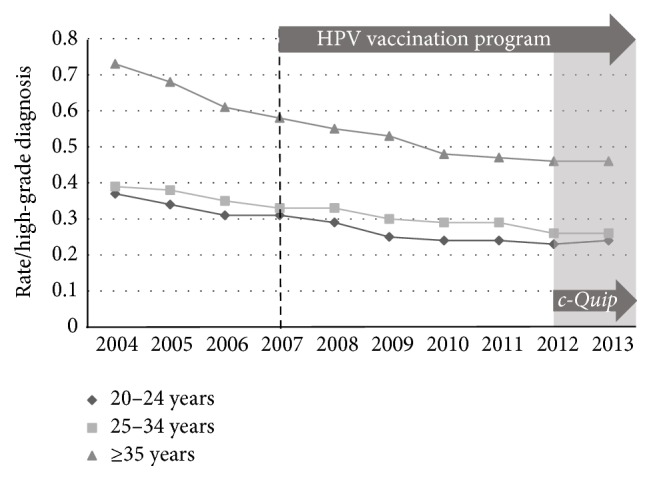
Medicare MBS billing data showing all excisional treatments (loop excision + cone biopsy) per histological diagnosis of high-grade change, age-stratified, for the period 2004–2013.

**Figure 7:**
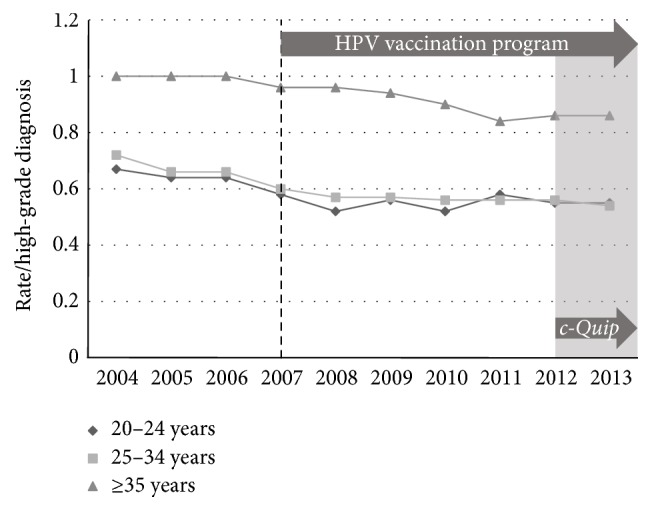
AIHW procedures data showing all excisional treatments (loop excision + cone biopsy) per histological diagnosis of high-grade change, age-stratified, for the period 2004–2013.

We examined the association between excisional treatment and the* c-Quip* program in two ways. Firstly the rate of all excisional treatments (loop excision + cone biopsy) per high-grade diagnosis (CIN2 + CIN3 + adenocarcinoma* in situ*) was calculated across the study period. Using this comparison, the smaller MBS dataset revealed falls across all three age-bands (Figure 6). The AIHW procedural dataset also revealed significant reductions in the rate for women in all age groups (Figure 7).

**Figure 8:**
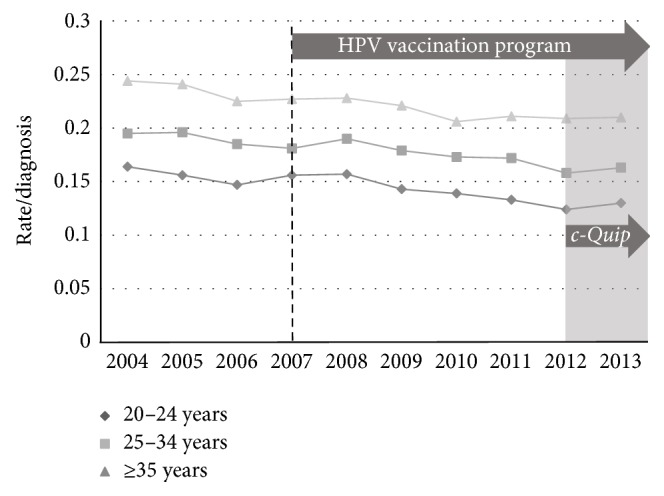
Medicare MBS billing data showing all loop excision per histological diagnosis of nonglandular dysplasia (LGEA + HGEA), age-stratified, for the period 2004–2013.

**Figure 9:**
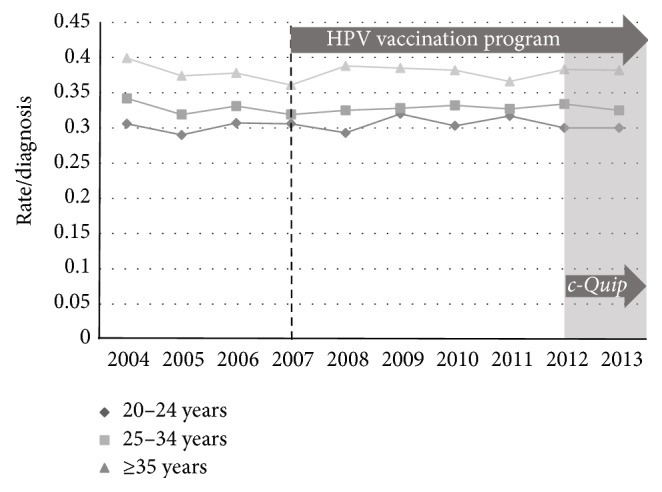
AIHW procedures data showing all loop excision per histological diagnosis of nonglandular dysplasia (LGEA + HGEA), age-stratified, for the period 2004–2013.

Secondly, to examine the rate of loop excision alone we calculated the rates using all nonglandular histological diagnoses (LGEA + HGEA) as the denominator in both datasets. In the MBS dataset there were falls in the rate in all three age groups (Figure 8). However, the* c-Quip* epoch was not associated with any change in the rate of loop excision in any age group: 20 to 24 years (*p* = 0.29); 25 to 34 years (*p* = 0.21); and 35 years or more (*p* = 0.34). In the AIHW dataset there was no change in the rate in any age group; the* c-Quip* epoch was not associated with any change (Figure 9).

## 4. Discussion

In Australia over the last decade there has been a decline in the incidence of low-grade squamous dysplasia across all age groups, particularly noticeable in women aged 20 to 24 years. This change predated introduction of the national vaccination program. The incidence of high-grade squamous dysplasia has also fallen in young women, with the fall in rate beginning well before any plausible effect from the vaccination program, but with slight but significant increases in older age groups. The rate of glandular abnormality remained unchanged in women aged 20 to 24 years and those aged 35 years or older, although there has been a significant increase in women aged 25 to 34 years. At a population level, the incidence rate of excisional treatments has fallen significantly in women aged less than 35 years. However, while the MBS dataset revealed small but significant reductions in the rate of loop excision per diagnosis of squamous dysplasia, the AIHW showed no significant changes. The* c-Quip* epoch was not associated with a fall in the rate of excisional treatment in any age group. A strength of this study is that it has used multiple national databases to build up a picture of the trends in excisional treatment of squamous and glandular dysplasia. However, it has not been possible to examine the use of ablation treatments (such as radical diathermy or laser).

The use of excisional treatment for cervical dysplasia is an important component of cervical cancer prevention, but we have been unable to identify any published studies examining trends in the incidence rate of excisional treatment in other countries. For this reason it is difficult to determine whether the national trends found in Australia are consistent with changes in clinical practice elsewhere in the world. It is noteworthy that the reduction in the use of excisional treatment in younger women began before the introduction of the National HPV Vaccination Program in 2007.

Perceptions of potential overuse of excisional treatment in nulliparous women, and indeed for young women whose family is not yet complete, prompted initiation of the* c-Quip* program [4]. Systematic reviews suggest that loop excision is associated with an increased risk of subsequent preterm birth [7] and an increased risk of miscarriage in the second trimester [8]. The risk for preterm birth appears to be increased after loop excision, irrespective of the histopathology being treated, and is highest after repeat procedures [9].

Young women diagnosed with high-grade cervical dysplasia are commonly affected by other confounding factors predisposing them to preterm birth [10]. Women who have undergone excisional treatment are more likely to have a shorter midtrimester cervical length [10]; however the question of whether depth or volume of tissue excised influences the risk of preterm birth remains unresolved. Some studies indicate a direct relationship [7] while others suggest the association may be a consequence of confounding and not a direct result of treatment [11]. Large excisions, thicker than 1.2 cm and larger than 6 cm, have been associated with a three times greater risk for preterm birth [12] and there appears to be an association between a short interval between excisional treatment and conception and adverse pregnancy outcomes [13, 14]. There does not appear to be any evidence that treatment for cervical intraepithelial neoplasia adversely affects fertility [8] or results in other adverse pregnancy outcomes [15].

The use of HPV vaccination programs in developed countries has been associated with significant population-level reductions in the incidence of high-grade squamous cervical dysplasia in young women. However, it remains unresolved whether this is a true result of immunization or reflects reduced uptake of screening [16]. A population study from Denmark, where participation in cervical screening is high, has revealed a reduction in the incidence of invasive squamous carcinoma in older women, with no changes in younger women as yet, and an increasing incidence of adenocarcinoma in young women [17, 18]. A similar study from the province of British Columbia in Canada examined the rates of cervical dysplasia before and after introduction of an HPV vaccination program and reported a significant reduction in high-grade squamous lesions in young women despite vaccine uptake levels below 70% [19]. Authors have suggested that, based on the changing pattern of cervical dysplasia in young vaccinated women, initial management of screen-detected abnormalities in young women should consist of cytologic and colposcopic surveillance in the first instance [20].

The national program to vaccinate young Australian women against high-risk strains of HPV has been widely embraced, with high levels of coverage with the full three doses in the target population. The program already has been associated with reductions in the prevalence of HPV vaccine-related infections in young women, a reduction in genital warts, and a reduction in high-grade cervical lesions in this age group [3]. The Australian National Cervical Screening Program is soon to change from cytology to primary screening for high-risk HPV, with a longer interscreening interval [21]. To ensure that the use of excisional treatment is appropriate, with lower rates for younger HPV-vaccinated women with a good prognosis and higher rates for older unvaccinated women, close surveillance, audit, and ongoing education will be required.
